# Long noncoding RNA MEG3, a potential novel biomarker to predict the clinical outcome of cancer patients: a meta-analysis

**DOI:** 10.18632/oncotarget.14987

**Published:** 2017-02-01

**Authors:** Xiangrong Cui, Xuan Jing, Chunlan Long, Jie Tian, Jing Zhu

**Affiliations:** ^1^ Pediatric Research Institute, Children's Hospital of Chongqing Medical University, Ministry of Education Key Laboratory of Child Development and Disorders, Chongqing, 400014, China; ^2^ China International Science and Technology Cooperation Base of Child Development and Critical Disorders, Chongqing, 400014, China; ^3^ Chongqing Key Laboratory of Pediatrics, Chongqing, 400014, China; ^4^ Cardiovascular Department (Internal Medicine), Children's Hospital of Chongqing Medical University, Chongqing, 400014, China; ^5^ Clinical Laboratory, Shanxi Province People's Hospital, Shanxi, 030000, China

**Keywords:** lncRNA, MEG3, clinical outcome, carcinoma, meta-analysis

## Abstract

Numerous studies have demonstrated that the expression level of maternally expressed gene 3 (MEG3) was lost in various cancers. Low expression of MEG3 is associated with an increased risk of metastasis and a poor prognosis in cancer patients. This meta-analysis investigated the association between MEG3 levels and distant metastasis (DM), lymph node metastasis (LNM), overall survival (OS), and recurrence-free survival (RFS) of cancer patients. A total of 536 participants from 9 articles were finally enrolled. The results showed a significant negative association between MEG3 levels and DM (OR = 2.16, 95% CI = 0.99–4.71, *P* = 0.05, fixed-effect), and it could also predict poor OS (HR = 0.43, 95% CI = 0.15–1.24, *P* = 0.006, fixed-effect) and RFS (HR = 0.52, 95% CI = 0.29–0.92, *P* = 0.02, fixed-effect) in cancer patients. In conclusion, this meta-analysis indicated that MEG3 might serve as a potential novel biomarker for indicating the clinical outcomes in human cancers.

## INTRODUCTION

Cancer has become one of the most important diseases threatening human health and life [1–3]. Exploring early diagnosis and treatment are critical for the research and clinical treatment of cancers [4, 5]. Recently, researchers focus on the molecular mechanism and new tumor biomarkers associated with tumor screening, diagnosis, prognosis, and evaluation of treatment efficacy [6–8]. However, the exact mechanism of cancers is still unknown. Therefore, to identify sensitive and specific biomarkers for prognosis of patients with cancers is urgently needed.

Long non-coding RNAs (lncRNAs) are a class of non-coding transcripts longer than 200 nucleotides [9, 10]. By comparing their expression of tumors and normal cells, lncRNAs are abnormally expressed in the various tumors, functioning as oncogenes or tumor suppressors [11–14].

Maternally expressed gene 3 (MEG3) is the first lncRNA to be found to have tumor suppressor function, which is expressed in many human normal tissues [15]. The lost expression of MEG3 has been found in many human tumors, such as bladder cancer [16], cervical carcinoma [17], hepatocellular cancers [15], and meningiomas [18]. In addition, hypermethylation of promoter or intergenic differentially methylated region (DMRs) upstream of MEG3 gene has been found to exert a vital role in the silence of MEG3 expression in tumors [19]. Moreover, MEG3 could inhibit cell proliferation in non-small lung cancer by inducing the expression of P53 [20, 21]. Together, lncRNA MEG3 may not only act as a potential therapeutic target, but also as a novel prognostic biomarker in cancer. However, no meta-analysis was been conducted assess the association between MEG3 and the survival of patients with cancers. Therefore, this meta-analysis evaluated the value of the MEG3 with tumor metastasis, progression, and survival.

## RESULTS

### Study characteristics

**Figure 1:**
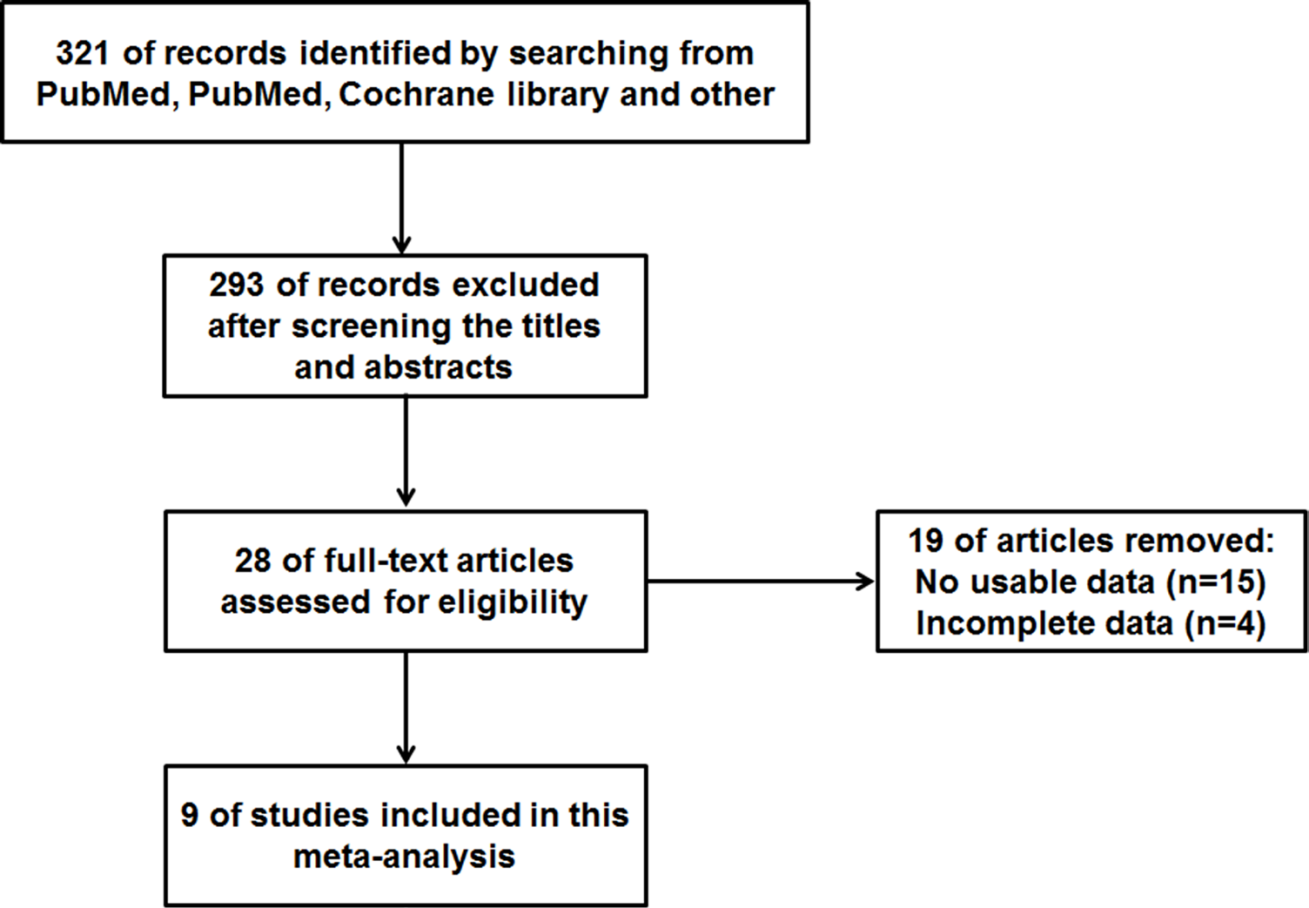
The flow diagram of this meta-analysis

As shown in Figure 1, we searched 321 articles in the databases. After screening the titles and abstracts, 28 full-text articles were assessed for eligibility. Then because of no usable data or incomplete data, 19 papers were excluded. As a result, a total of 9 articles were in the current meta-analysis [22–30]. Eight different types of cancer were evaluated in this meta-analysis, with 1 non-small cell lung cancer (NSCLC), 2 gastric cancer (GC), 1 tongue squamous cell carcinoma (TSCC), 1 non-functioning pituitary adenomas (NFPAs), hepatocellular carcinoma (HCC), 1 osteosarcoma, 1 prostate cancer (PC), 1 bladder cancer (BC). In these included studies, the level of MEG3 expression was determined in collected tumor tissues.

Table 1 summarized the main characteristics of the included 9 studies ranging 2013 to 2016. 9 studies enrolling 536 participants, with a maximum sample size of 80 and a minimum sample size of 21 patients. Because the cut-off definitions were various, the cut-off values were different in these studies. Not all studies examined both OS and RFS, because most of the studies were retrospective cohort studies; 6 studies investigated the association between MEG3 and OS [22–26, 30], while 2 studies assessed the association between CCAT2 and RFS [25, 29]. Meanwhile, in the including 9 studies, 5 articles performed the relationship between the expression of MEG3 and gender [23, 25–27, 30], 3 articles demonstrated that MEG3 were correlated with lymph node metastasis (LNM) [23, 28, 30], and 3 were on distant metastasis DM [23, 26, 30].

**Table 1:** Characteristics of the included studies

First author	Year	Country	Tumor type	Sample	Reference	Detection method	Sample size	Outcome	Cut-off value
Kaihua Lu [22]	2013	China	NSCLC	tissue	GAPDH	q-PCR	44	OS	0.27
Ming Sun [23]	2014	China	GC	tissue	GAPDH	q-PCR	72	OS	0.377-fold
Lingfei Jia [24]	2014	China	TSCC	tissue	β-actin	q-PCR	76	OS	mean
Zhenye Li [27]	2014	China	NFPAs	tissue	GAPDH	q-PCR	52	—	median
Han Zhuo [25]	2015	China	HCC	tissue	GAPDH	q-PCR	72	OS, RFS	median
Zhizhong Tian [26]	2015	China	osteosarcoma	tissue	GAPDH	q-PCR	64	OS	median
Gang Luo [28]	2015	China	PC	tissue	GAPDH	q-PCR	21	—	NA
Feifei Meng [30]	2016	China	GC	tissue	GAPDH	q-PCR	55	OS	NA
Weili Duan [29]	2016	China	BC	tissue	GAPDH	q-PCR	80	RFS	median

NSCLC: non-small cell lung cancer, GC: gastric cancer (GC), TSCC: tongue squamous cell carcinoma, NFPAs: non-functioning pituitary adenomas, HCC: hepatocellular carcinoma, PC: prostate cancer (PC), BC: bladder cancer; OS: overall survival; RFS: recurrence-free survival.

### Association between lncRNA MEG3 and clinicopathological characteristics

**Figure 2:**
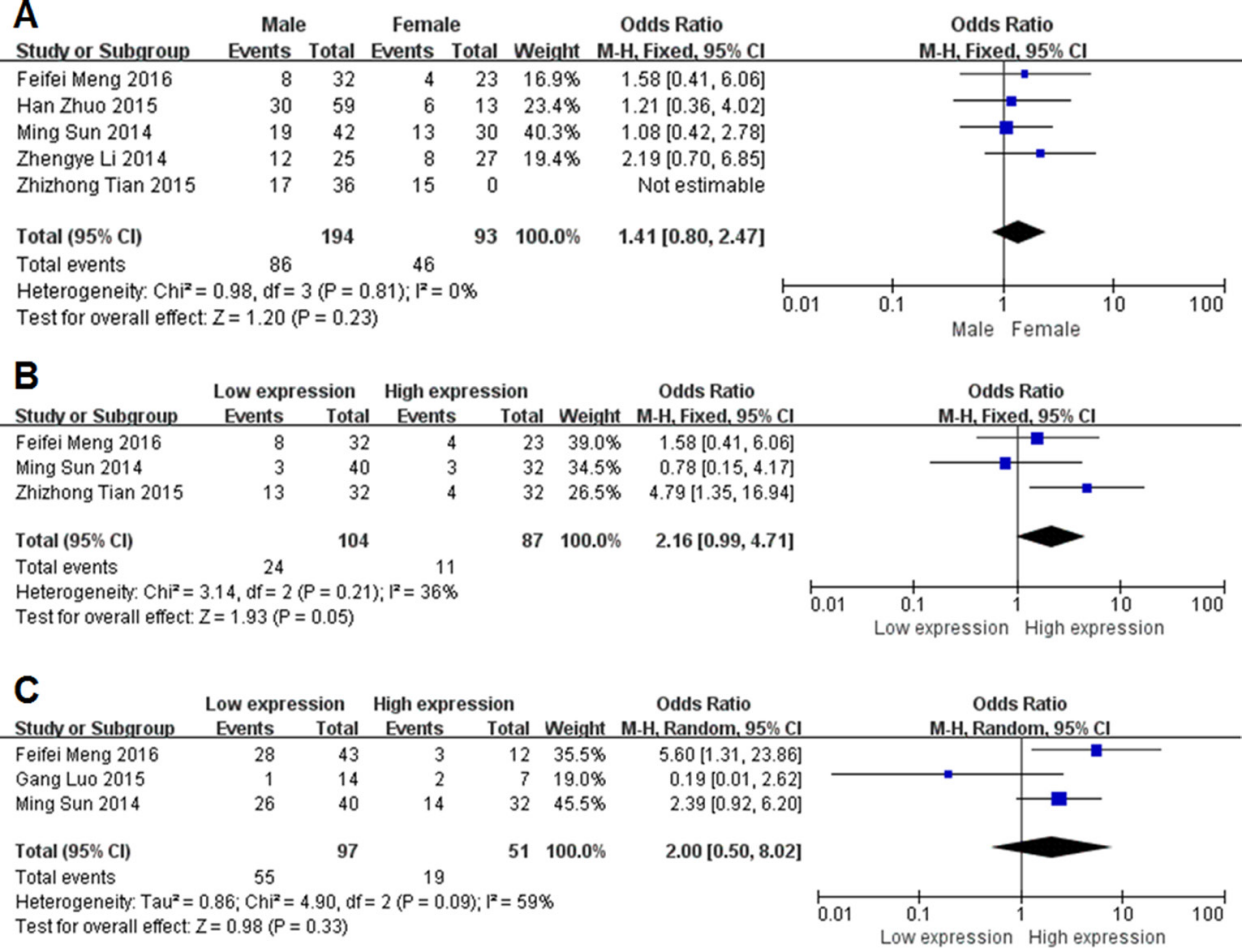
Forest plot for the association between lncRNA MEG3 and Clinicopathological characteristics (**A**) gender; (**B**) distant metastasis; (**C**) lymph node metastasis.

As shown in Figure 2, we performed a meta-analysis to evaluate the relationship between the transcription levels of MEG3 and clinicopathological characteristics of patients with cancer. Our results demonstrated that the expression levels of MEG3 were not associated with the gender of patients (OR = 1.41, 95% CI = 0.80–2.47, *P* = 0.23, fixed-effect) (Figure 2A). Three studies reported the relation between MEG3 and DM. The fixed-effects model was adopted as the significant heterogeneity (I^2^ = 36%, *P* = 0.21). Compared with high MEG3 expression group, low MEG3 expression group had a statistic significant elevated DM rate (OR = 2.16, 95% CI = 0.99–4.71, *P* = 0.05, fixed-effect) (Figure 2B). Unfortunately, there was no correlation in LNM (OR = 2.00, 95% CI = 0.50–8.02, *P* = 0.33, random-effect) (Figure 2C).

### Association between lncRNA MEG3 and prognosis

**Figure 3:**
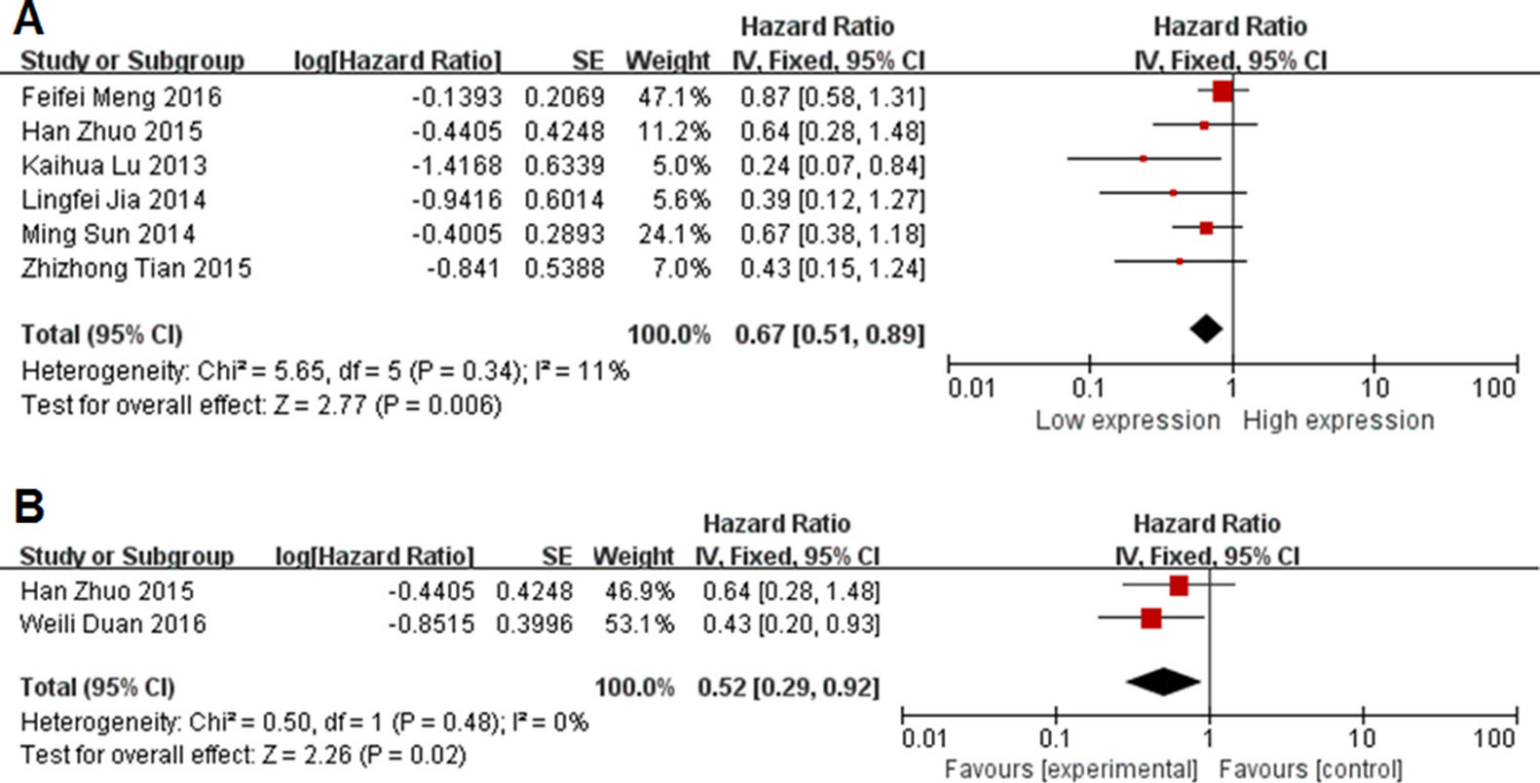
Forest plot for the association between lncRNA MEG3 and prognosis (**A**) overall survival; (**B**) recurrence-free survival.

We analysis pooled HRs of two group. Six studies with 384 patients were included in this meta-analysis of OS (Figure 3A). Because of no significant heterogeneity (*I*^2^ = 11%, *P* = 0.34), the fixed-effects model was chosen to estimate the pooled HRs with corresponding 95% CIs. Compared with high MEG3 expression group, low MEG3 expression group had a statistic significant reduced OS (HR = 0.43, 95% CI = 0.15–1.24, *P* = 0.006, fixed-effect) (Figure 3B). Low MEG3 expression correlated with a worse survival. Due to significant heterogeneity (*I*^2^ = 0%, *P* = 0.48), fixed-effects model was used. Analysis showed a pooled HR = 0.43 (95% CI = 0.29–0.92, *P* = 0.02). Compared with high MEG3 expression group, low MEG3 had a statistic significant reduced RFS.

### Publication bias and sensitivity analysis

**Figure 4:**
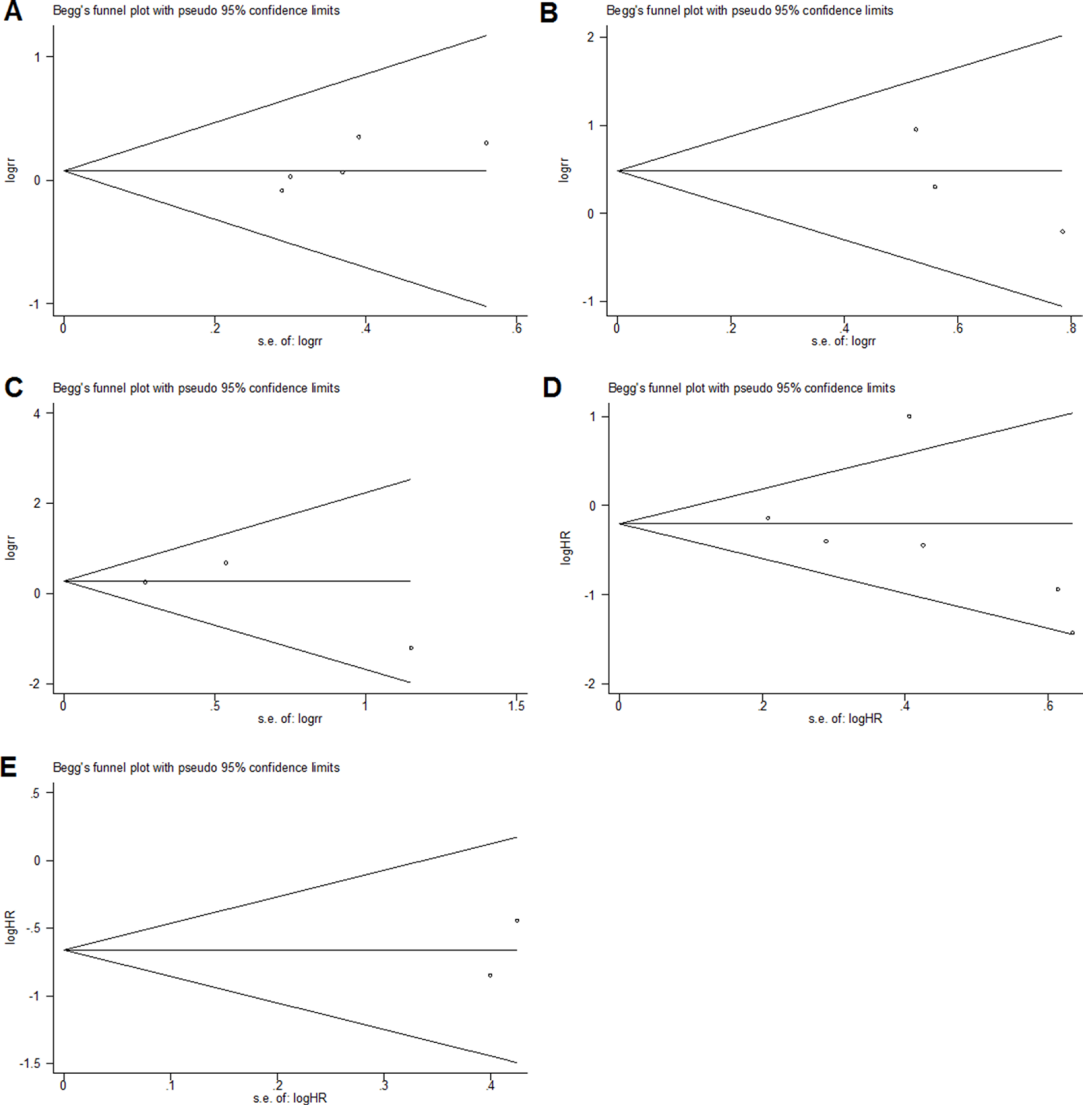
Begg's test for publication bias (**A**) gender; (**B**) distant metastasis; (**C**) lymph node metastasis; (**D**) overall survival; (**E**) recurrence-free survival.

**Figure 5:**
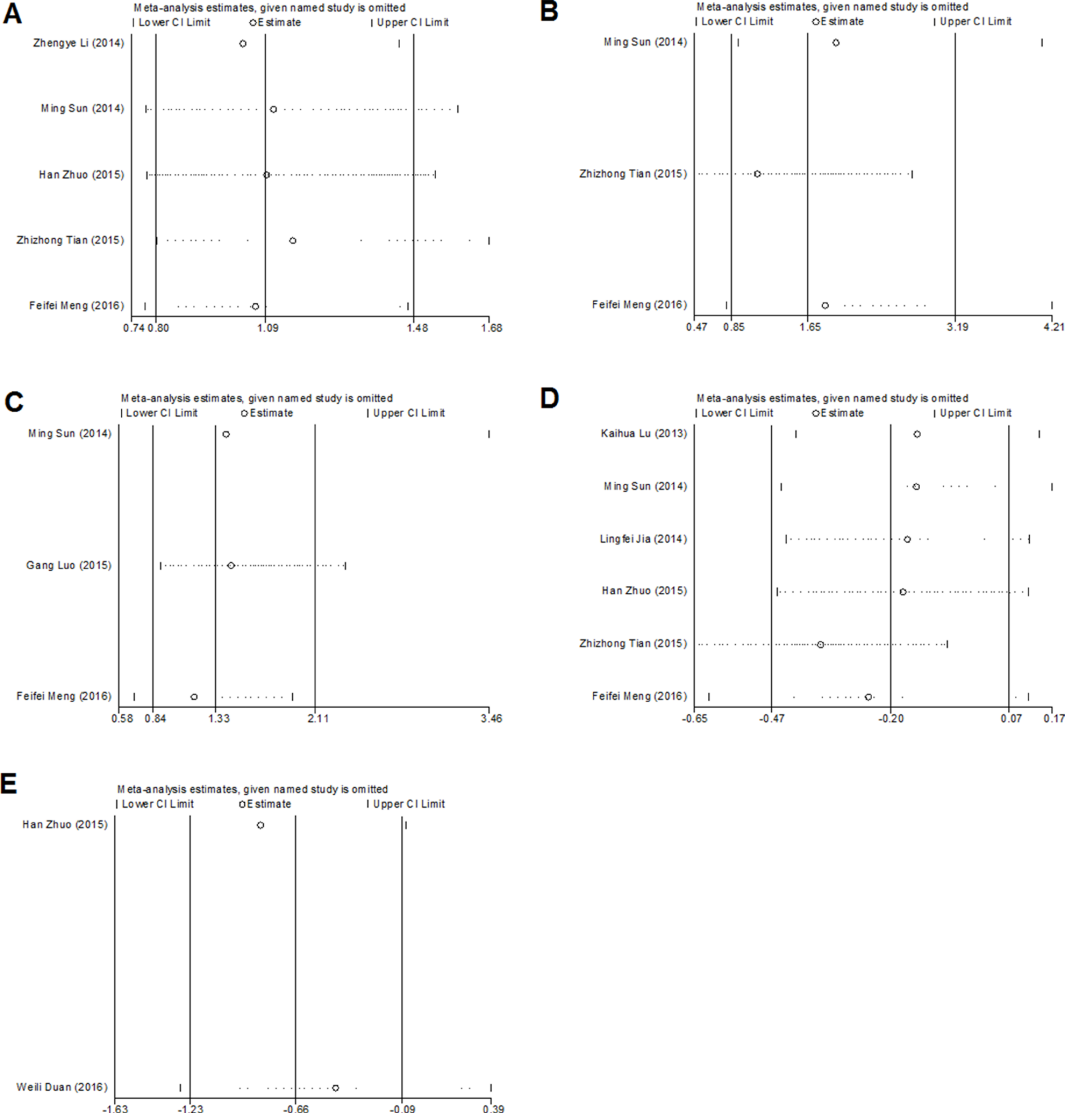
Sensitivity analyses of the studies (**A**) gender; (**B**) distant metastasis; (**C**) lymph node metastasis; (**D**) overall survival; (**E**) recurrence-free survival.

As shown in Figure 4, Begg's test was used to perform the publication bias, respectively. In our meta-analysis, Begg's test indicated there were no publication bias in all groups, due to all the values of *P* > 0.05. Meanwhile, we used Stata11.0 software to evaluate sensitivity analysis to assess whether the individual studies affected the overall results. The results suggested that individual study had little influence on our final results (Figure 5), and demonstrated that our analysis was relatively stable and credible.

## DISCUSSION

Along with the research of human genomics, it is found that only 2% of the genomic sequences are translated into proteins, and most of which are transcribed into non-coding RNA [31]. LncRNAs were once considered to be the noise of genome transcription without biological function. However, recent studies have indicated that lncRNAs are closely related to many diseases, such as neurodegenerative diseases [32], cardiovascular diseases [33], rheumatoid diseases [34] and so on. Meanwhile, abnormal expressions of lncRNAs have been found in tumor tissues, which play important role in the carcinogenesis and aggressive progression of human malignancies [35]. These finding suggested that the potential role of lncRNAs should be further investigated.

MEG3 is an imprinted gene belonging to the DLK1-MEG3 locus located on chromosome 14q32.3 which is the first lncRNA to be found with tumor suppressor function [36]. Previous studies have showed that MEG3 was expressed in brain [36], pituitary [37], ovary [38] and other normal tissues, while the expression was reduced or even lost in a variety of tumor cell lines [39]. Meanwhile, overexpression of MEG3 can inhibit the proliferation of tumor cell lines, which indicates that it plays a role of tumor suppressor genes. In non-small cell lung cancer, Lu et al. have showed that lower expression of MEG3 had advanced clinical features and poor prognosis [22]. In 2015, Yin et al. [40] demonstrated that MEG3 was remarkably decreased in colorectal cancer tissues, comparing adjacent normal control tissues, and the lower expression of MEG3 could promote cells proliferation *in vitro*. Sun et al. [41] reported that down-regulated MEG3 enhanced the cell proliferation and migration *in vitro* and increased tumor growth and metastasis in gastric cancer. Thus, MEG3 could be considered as a potential prognostic factor for various cancers. In our meta-analysis, we assessed the prognostic role of MEG3 in cancers. Our results indicated that lower expressions of MEG3 represented a risk factor for OS in cancers (HR = 0.43, 95% CI = 0.15–1.24, *P* = 0.006, fixed-effect). Furthermore, we found that there was significantly relationship between MEG3 and RFS (95% CI = 0.29–0.92, *P* = 0.02). From these results, lncRNA MEG3 could be as biomarker for the prognosis of cancers. However, further large-scale studies should be conducted.

It has been reported that MEG3 can play important role in inhibiting cancer through a variety of ways, which related to DNA methylation, P53 pathway, Rb pathway and so on [42]. Sun L et al. suggested that lncRNA EWSAT1 can enhance osteosarcoma cell growth and metastasis through suppression of MEG3 expression [43]. Besides, MEG3 plays important role in the epigenetic regulation of epithelial-mesenchymal transition promotes in lung cancer. In this meta-analysis, we evaluate the association between expression levels of MEG3 and cancer. We found that lower levels of MEG3 were more prone to lead to DM (OR = 2.16, 95% CI = 0.99-4.71, *P* = 0.05). Unfortunately, there was no correlation in LNM (OR = 2.00, 95% CI = 0.50–8.02, *P* = 0.33); insufficient sample size was the possible reason for the different results in this meta-analysis. Therefore, further studies should be done with larger sample sizes.

It should be stressed the limitations in our analysis. Most studies reported positive results, but those with negative results were generally less likely to be published. In addition, studies included in the meta-analysis most came from People's Republic of China, which might affect the results. Finally, there were insufficient data to fully confirm the association between MEG3 and clinicopathological characteristics, which needs more studies. Therefore, the results of this meta-analysis should be confirmed in future studies.

In conclusion, despite the limitations described above, our meta-analysis reveals that the depressed expression of lncRNA MEG3 is significantly associated with DM, OS and RFS in patients with diverse cancers and could be a potential prognostic marker for cancers. However, large-scale and comprehensive researches were needed to illuminate our results. Well-designed studies related to specific cancer types and large sample sizes are needed to confirm the prognostic value of decreased lncRNA MEG3 in various cancers.

## MATERIALS AND METHODS

### Literature search strategies

We searched the databases PubMed, Cochrane library, Chinese National Knowledge Infrastructure, and Chinese Wan Fang database for studies published up to November 2016 to obtain relevant articles for the meta-analysis. The search strategy used both medical subject heading terms and free-text words to increase the sensitivity of the search. The keywords for the search were as follows: “MEG3 and cancer”, “long non-coding RNA MEG3”, “lncRNA MEG3”, “MEG3”. There was no language restriction. Meanwhile, reference lists of relevant articles were also reviewed to identify potential eligible papers.

### Inclusion and exclusion criteria

In this meta-analysis, eligible studies had to meet the following standards: 1)cohort design, 2) articles investigating the relation of MEG3 and cancer patients; 3) the expression levels of MEG3 in primary tumor tissues were measured, 4) sufficient original data for calculating odds ratios (ORs), hazard ratios (HRs) and their 95% confidence interval (95% CI). If the articles only provided survival curves without offering HR and 95% CI directly, appropriate data were extracted from the survival curves using Engauge Digitizer 4.1 software and the logHR and selogHR calculated according to Tierney et al. [44]. If there were duplicated data, we chose the most complete data or the most recent one. Exclusion criteria were as follow: 1) studies without usable or insufficient data, 2) case reports, 3) letters and conference abstracts.

### Data extraction

Two investigators extracted and reviewed relevant data from the eligible studies independently, including first author, year of publication, country, site of cancer, method, case number, cut-off value. If there were disagreements, a consensus was reached by the third investigator.

### Statistical analysis

Odds ratios (ORs) and 95% CI were used to evaluate the relationship between MEG3 and in these inclusive articles. The features included gender, distant metastasis (DM), and lymph node metastasis (LNM). Meanwhile, HRs and 95% CIs were used to assess the association between MEG3 and cancer prognosis (Relapse-free survival and overall survival). We used Revman5.3 Software (Revman, the Cochrane Collaboration) to perform the meta-analysis and evaluate heterogeneity between studies by Cochrane *Q*-test and *P*-values. If heterogeneity was present (I^2^ ≥ 50% or *P* ≤ 0.05), random-effect model was used to calculate pooled HRs or ORs. If not, the fixed-effect model was more appropriate [45, 46]. The Stata11.0 Software (Stata, College Station) was performed to evaluate the sensitivity and publication bias of the studies. Publication bias was evaluated by Begg's test, *P* < 0.05 was considered statistically significant.
